# Intralesional Treatment of Non-American Cutaneous Leishmaniasis with Multiple Lesions

**DOI:** 10.4269/ajtmh.24-0667

**Published:** 2025-01-28

**Authors:** Clarissa Pieri, Stephen L. Walker

**Affiliations:** ^1^ University of Liverpool, Liverpool, United Kingdom;; ^2^ Department of Dermatology, University College London Hospitals NHS Foundation Trust, London, United Kingdom;; ^3^ Hospital for Tropical Diseases, University College London Hospitals NHS Foundation Trust, London, United Kingdom;; ^4^ Faculty of Infectious and Tropical Diseases, London School of Hygiene and Tropical Medicine, London, United Kingdom

**Figure 1.**
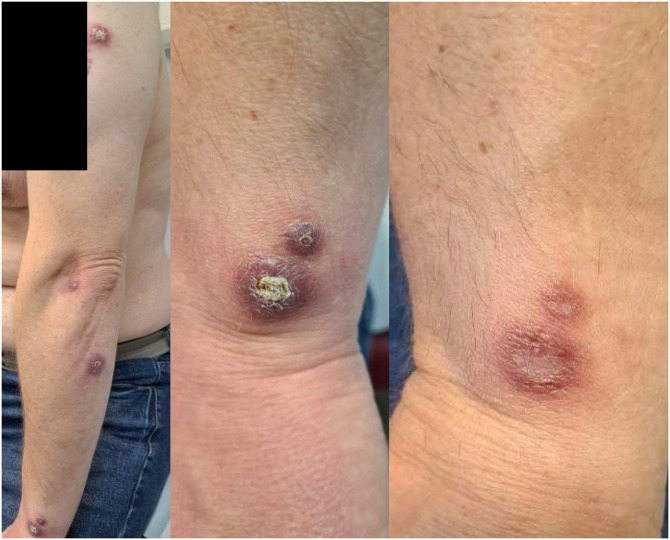
Distribution of lesions of cutaneous leishmaniasis with close up view of wrist lesions before and after treatment at day 90.

A 44-year-old male developed five ulcerated plaques on the left upper limb (Figure 1) while in Sicily, Italy. The size of lesions varied between 1 and 2.5 cm 1 year before presentation at the Hospital for Tropical Diseases, London. There was no lymphadenopathy, lymphangitis, or subcutaneous induration. He was HIV uninfected. The ulcerated lesions had not responded to separate courses of oral clarithromycin, doxycycline, or cotrimoxazole. A skin biopsy showed granulomatous inflammation, and *Leishmania donovani* complex DNA was detected, confirming the diagnosis of cutaneous leishmaniasis (CL).

The treatment options for CL were discussed, including conservative management, intralesional (IL) treatment with meglumine antimoniate (MA), miltefosine, intravenous MA, or liposomal Amphotericin B. The number of CL lesions on our patient raised an interesting issue with respect to three published CL treatment guidance documents, which advise systemic therapy for individuals with “complex” CL.^1^

Our patient met the criteria for “simple” rather than “complex” CL; he was immunocompetent, and the causative *Leishmania* species was unlikely to be associated with mucosal disease and the location of the lesions. However, all three publications categorized our patient as “complex” because he had five CL lesions. Two recommend systemic therapy if there are four or more CL lesions, and the other publication recommends systemic therapy if there are more than four of “substantial size,” defined as greater than 1 cm.^1^^–^^3^ The other two guidance documents use different lesion sizes (3 and 4 cm) for categorizing CL as complex (independent of the number of lesions).^2^^,^^3^

The patient wished to have active treatment given the prolonged duration of the skin lesions and continued inflammation. He expressed a preference for local treatment rather than systemic treatment because of the reduced risk of adverse effects.^4^

Weekly IL MA (300 mg/mL) was administered intradermally to all lesions on five occasions initially and was well tolerated. The volume of MA required for infiltration decreased at each administration. Six milliliters were initially required, and 1.5 mL were required for two lesions at the final treatment session. All lesions flattened and re-epithelialized with scarring when reviewed at day 90.

The three guidance documents are extremely useful for physicians managing individuals with CL, particularly those who may be unfamiliar with the disease. Choosing the best CL therapy requires affected individuals to be supported to make informed decisions about their care. This may mean deviating from expert recommendations, particularly where the evidence for such recommendations is lacking.
